# The Impact of Fructose Consumption on Human Health: Effects on Obesity, Hyperglycemia, Diabetes, Uric Acid, and Oxidative Stress With a Focus on the Liver

**DOI:** 10.7759/cureus.70095

**Published:** 2024-09-24

**Authors:** Baharuddin Baharuddin

**Affiliations:** 1 Department of Medical Science, University of Surabaya, Surabaya, IDN

**Keywords:** insulin resistance, uric acid, oxidative stress, nafld, type 2 diabetes, obesity, excessive fructose intake, human health

## Abstract

Excessive fructose consumption, primarily through processed foods and beverages, has become a significant public health concern due to its association with various metabolic disorders. This review examines the impact of fructose on human health, focusing on its role in obesity, insulin resistance, hyperglycemia, type 2 diabetes, uric acid production, and oxidative stress. Fructose metabolism, distinct from glucose, predominantly occurs in the liver, where it bypasses normal insulin regulation, leading to increased fat synthesis through de novo lipogenesis. This process contributes to the development of non-alcoholic fatty liver disease and elevates the risk of cardiovascular disease. Furthermore, fructose-induced adenosine triphosphate depletion activates purine degradation, increasing uric acid levels and exacerbating hyperuricemia. The overproduction of reactive oxygen species during fructose metabolism also drives oxidative stress, promoting inflammation and cellular damage. By synthesizing recent findings, this review underscores the importance of regulating fructose intake, implementing public health policies, and adopting lifestyle changes to mitigate these adverse effects.

## Introduction and background

Processed foods and beverages are increasingly associated with a heightened risk of obesity, hyperglycemia, type 2 diabetes, elevated uric acid levels, and oxidative stress in humans. In recent decades, the rising daily consumption of fructose has become a primary concern due to its significant negative impact on human health. The number and variety of food and beverage products containing fructose continue to increase [1-3], primarily through the use of high-fructose corn syrup (HFCS). Furthermore, the lack of regulation and control at the state level facilitates the industry’s ability to add excessive amounts of sweeteners [4-6]. This situation presents a serious problem at the global population level, as excessive consumption can adversely affect various organs and lead to metabolic disorders in the human body.

One organ significantly impacted by excessive fructose consumption is the liver, primarily due to de novo lipogenesis. While there are various partial findings in fructose research, comprehensive reviews remain limited, despite their importance in understanding the overall impact of fructose. Given the emergence of numerous new findings in the field, it is essential to conduct studies that examine the latest research on the effects of fructose consumption on various metabolic diseases, particularly its influence on liver health.

## Review

Absorption and metabolism of fructose in the body

After consumption, fructose is absorbed in the small intestine using transporters such as glucose transporter 2 (GLUT2) and glucose transporter 5 (GLUT5) [7,8]. Disruption in the expression of fructose transporters, particularly GLUT5, can lead to impaired absorption of fructose in the small intestine [9]. GLUT5 functions and works on the apical intestinal tissue area, whereas GLUT2 works on the basolateral area to ensure fructose is inside circulation. Subsequently, the circulatory system distributes fructose to all body tissues, including the liver.

**Figure 1:**
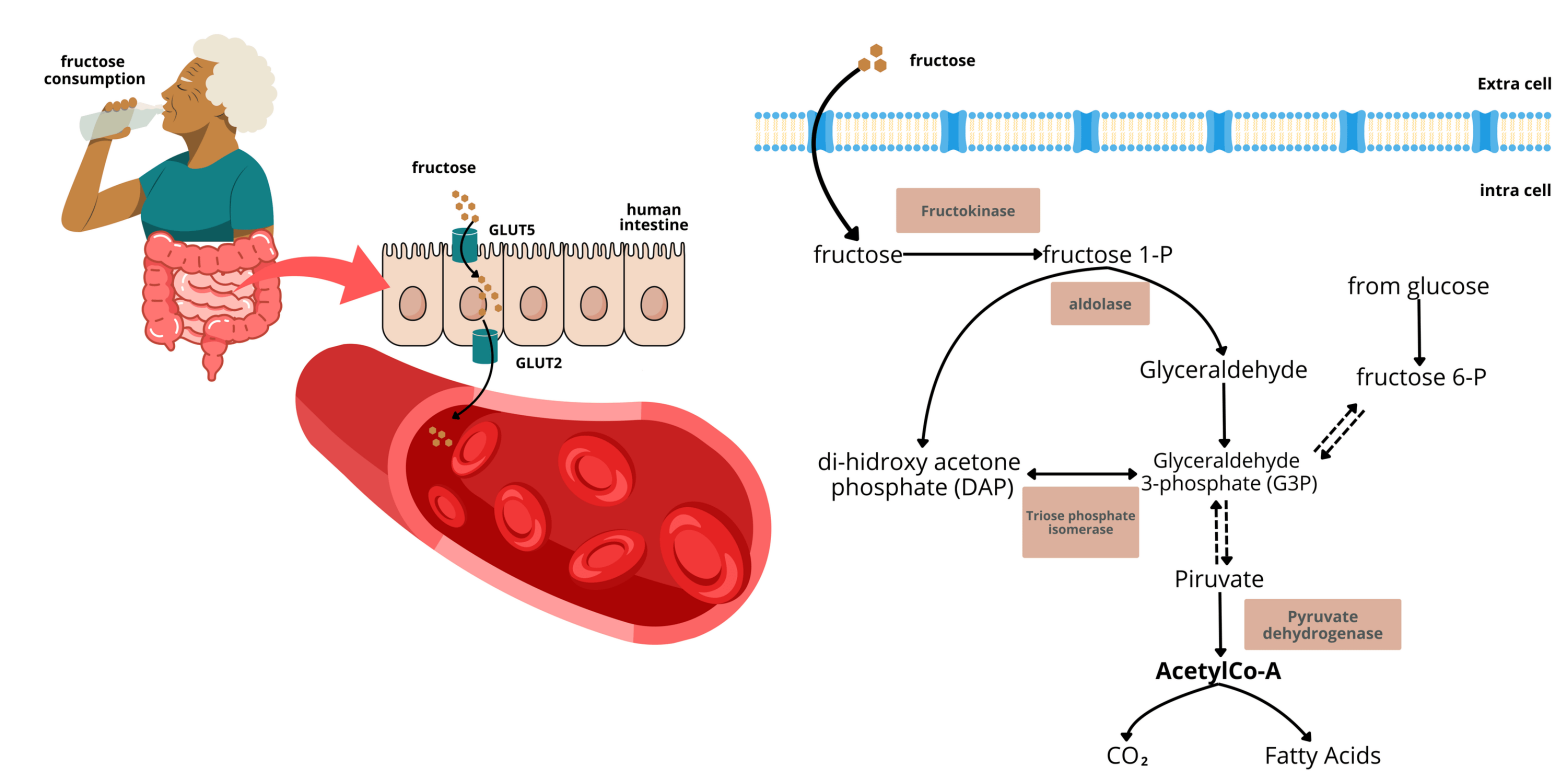
Absorption and metabolism of fructose in the cell Fructose 1-P: fructose 1-phosphate; GLUT2: glucose transporter 2; GLUT5: glucose transporter 5

The stages of the absorption process and the initial metabolism of fructose in the human digestive tract are illustrated in Figure 1.

In the subsequent stage, most fructose is metabolized in the liver [10]. Under normal conditions, fructose metabolism supports glycogen storage and de novo lipogenesis, which help maintain energy reserves. However, excessive daily fructose consumption can render this metabolic process inefficient. This inefficiency leads to energy depletion due to high adenosine triphosphate (ATP) utilization, along with the formation of uric acid, inflammation, liver fibrosis [11], and non-alcoholic fatty liver disease (NAFLD). The depletion of ATP occurs because fructose metabolism utilizes a unique pathway that is not subject to feedback control (non-insulin-dependent) [12,13]. Additionally, the metabolic pathway of fructose has a distinctive feature: it can bypass the step of converting to fructose-1-phosphate directly through the action of fructokinase (Figure 1). This differs from glucose metabolism, which is well-regulated by the body through insulin and proceeds stepwise through the glycolysis pathway.

Numerous enzymes contribute to fructose metabolism, prompting global researchers to explore this enzyme activity. Enzymes involved in the conversion of fructose to acetyl CoA are of particular interest. For example, fructokinase and aldolase B are crucial enzymes in fructose metabolism and are primarily studied (Table 1) [14].

**Table 1:** List of enzymes involved in fructose metabolism and related pathways

Enzyme	Main function	Class
Fructokinase	Catalyzes the conversion of fructose to fructose-1-phosphate	Transferases
Ketohexokinase	Catalyzes the phosphorylation of fructose to produce fructose-1-phosphate	Transferases
Aldolase B	Cleaves fructose-1-phosphate into dihydroxyacetone phosphate and glyceraldehyde	Lyases
Triose phosphate isomerase	Converts dihydroxyacetone phosphate into glyceraldehyde-3-phosphate	Isomerases
Glycerol dehydrogenase	Catalyzes the reduction of glyceraldehyde to glycerol-3-phosphate	Oxidoreductases
Glycerol kinase	Phosphorylates glycerol to glycerol-3-phosphate	Transferases
Triose kinase	Converts glyceraldehyde into glyceraldehyde-3-phosphate	Transferases
Pyruvate kinase	Catalyzes the conversion of phosphoenolpyruvate into pyruvate	Transferases
Pyruvate dehydrogenase	Converts pyruvate into acetyl-CoA through oxidative decarboxylation	Lyases

On the other hand, the body has the ability to maintain a certain balance of blood sugar levels. Interestingly, fructose can also be produced endogenously from glucose, particularly in pathological conditions such as kidney disease, diabetes, cardiac hypertrophy, and dehydration. Numerous studies have demonstrated that excessive fructose consumption correlates with an increased incidence of type 2 diabetes worldwide [15].

Fructose and obesity

High fructose consumption has been shown to cause insulin resistance in the liver and other tissues, ultimately contributing to obesity [16]. Additionally, the development of visceral fat in the human body poses a significant risk for visceral obesity [14]. Fructose is uniquely metabolized in the liver, where it is rapidly converted into glucose, glycogen, lactate, and fat. This rapid conversion occurs as fructose is transformed into fructose 1-phosphate by fructokinase or ketohexokinase, bypassing the hexokinase step [2,17]. As a result, fructose metabolism within the glycolysis pathway becomes more streamlined. An increase in fructose 1-phosphate levels subsequently stimulates the production of glyceraldehyde 3-phosphate [18].

**Figure 2:**
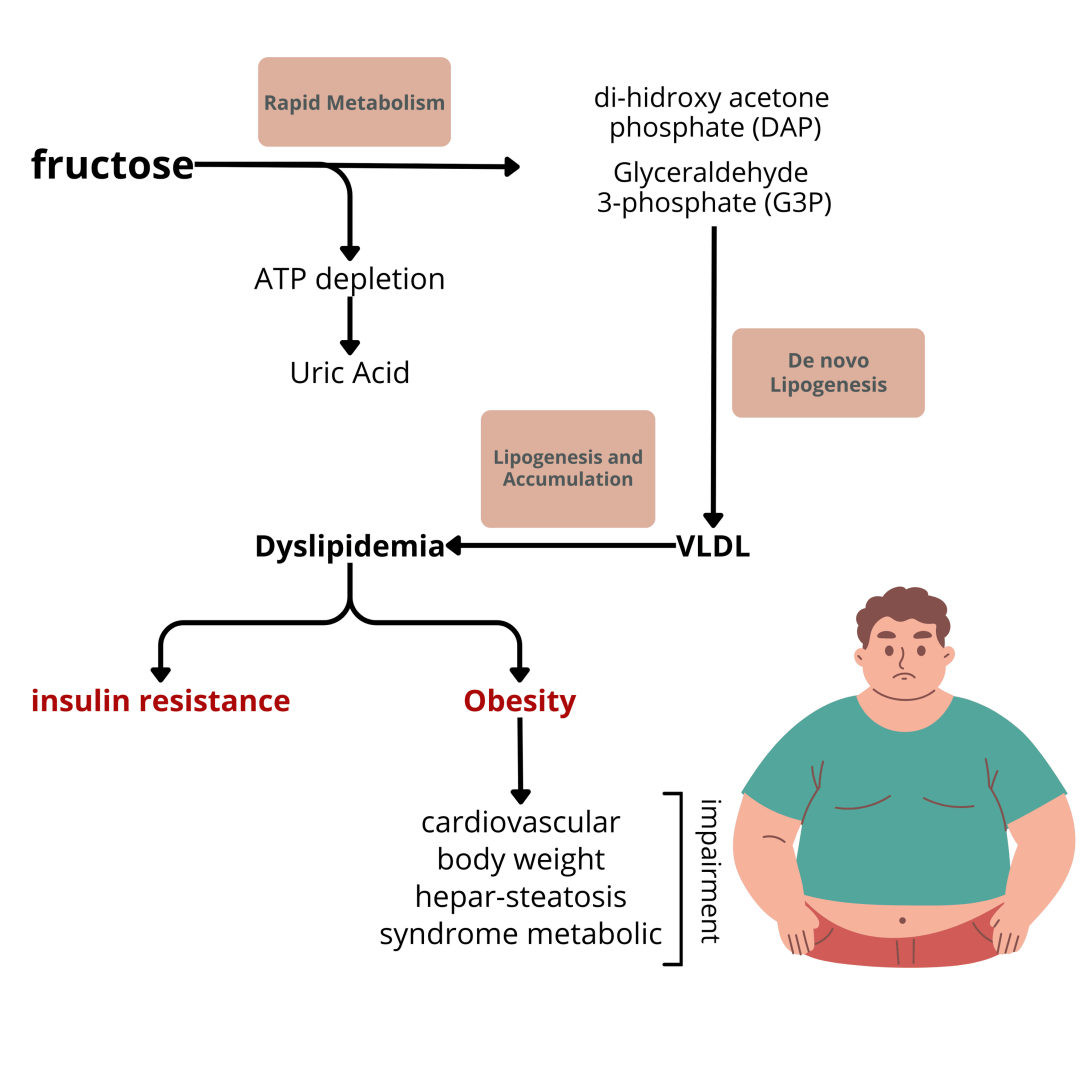
Mechanism by which fructose consumption contributes to obesity and related impairment ATP: adenosine triphosphate; VLDL: very low-density lipoprotein

The mechanism by which fructose consumption leads to obesity and related impairments is illustrated in Figure 2.

The subsequent sequence stimulates the production of pyruvic acid and acetyl-CoA. The formation of acetyl-CoA molecules in the mitochondria serves as a central point in the metabolism of fructose into fat, as this molecule acts as a precursor for converting non-fat sources into fat. This process can ultimately lead to a progressive accumulation of fat within liver cells and an increase in blood triglyceride levels, thereby raising the risk of obesity (Figure 2). While this pathway is not the primary mechanism driving the pathological effects of fructose, it can significantly contribute to the development of metabolic diseases associated with obesity [13,19]. Additionally, it is important to note that the mechanisms by which fructose leads to obesity remain a topic of debate, as they are progressive and require time for accumulation. Consequently, results from short-duration studies, particularly in animal models, may not yield significant findings.

In the context of liver metabolism and fatty deposition, fructose is closely linked to the progressive stimulation of de novo lipogenesis and the development of NAFLD. The risk of steatosis from high doses over short durations and progressive obesity from low doses over extended periods presents significant challenges in studying the effects of fructose consumption. To better understand the impact of fructose intake at the population level on the incidence of NAFLD, large-scale observational studies are essential [13,20,21]. This need for extensive research underscores the complexity of evaluating fructose consumption and its implications for public health.

Fructose, hyperglycemia, and type 2 diabetes

Examining the metabolic pathway reveals that fructose consumption can stimulate hyperglycemic conditions, a process that is progressive and time-dependent. Excessive intake of fructose can lead to hyperglycemia, or uncontrolled elevation of blood sugar levels, because fructose does not stimulate insulin production in the same way that glucose does [22,23]. Prolonged periods of hyperglycemia can, in turn, trigger the development of type 2 diabetes.

**Figure 3:**
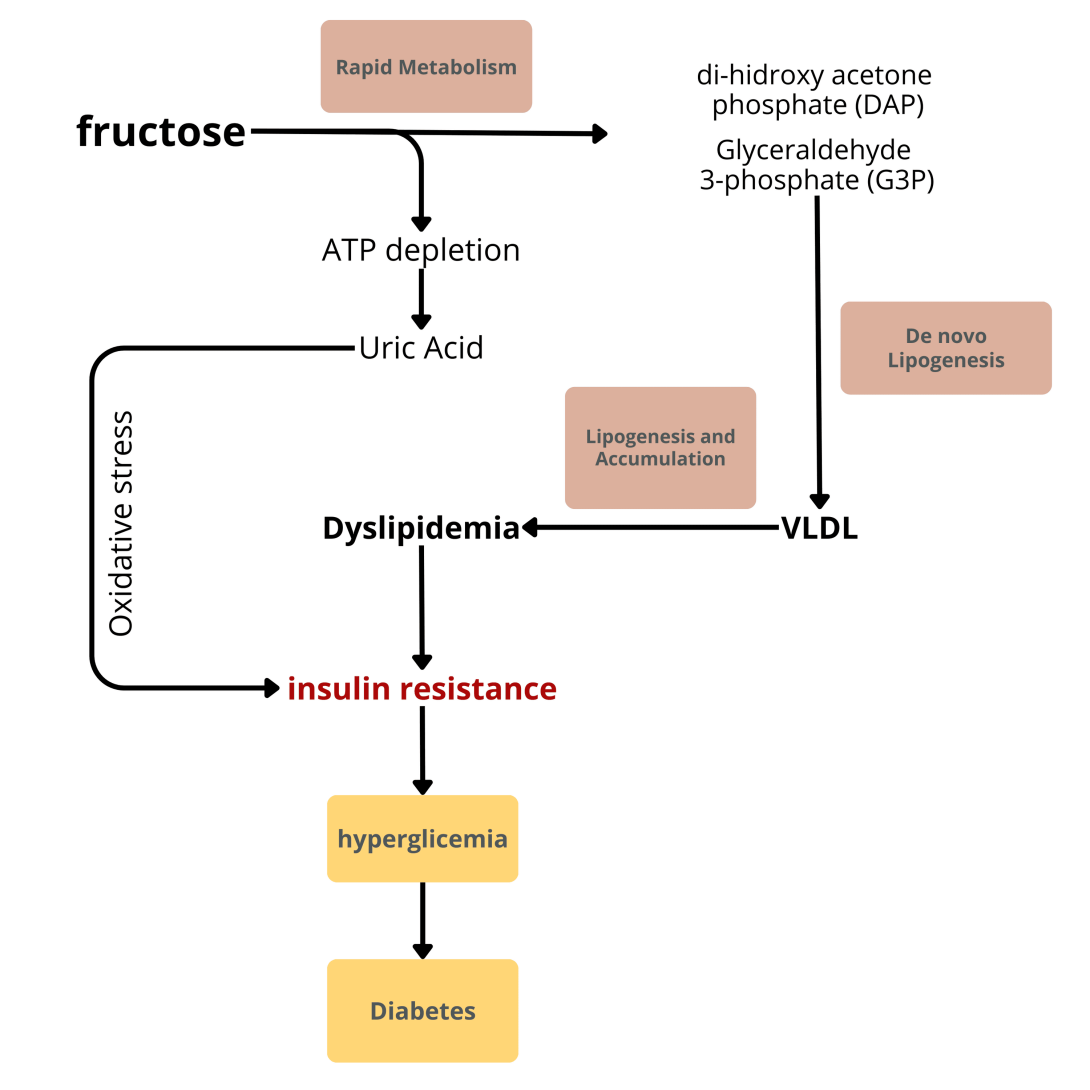
Mechanism by which fructose contributes to hyperglycemia and type 2 diabetes ATP: adenosine triphosphate; VLDL: very low-density lipoprotein

The mechanism through which fructose contributes to hyperglycemia and the onset of type 2 diabetes is illustrated in Figure 3.

Unlike glucose, which is absorbed by various tissues, fructose is primarily metabolized in the liver. In this organ, fructose is rapidly converted into fructose-1-phosphate without insulin regulation. This process saturates the glycolytic pathway in the liver, leading to a significant increase in acetyl-CoA production. This acetyl-CoA can then be utilized for fat synthesis or glucose formation [24-26]. The rapid metabolism of fructose stimulates dihydroxyacetone phosphate and glyceraldehyde-3-phosphate (Figure 3). Increased de novo lipogenesis in the liver enhances the production of lipoproteins, particularly very low-density lipoprotein. This rise in lipogenesis and fat deposition can lead to dyslipidemia, which may subsequently manifest as insulin resistance. Over time, this condition can progress to type 2 diabetes.

Studies have shown that patient adherence to medication is the most significant factor influencing blood sugar control in individuals with type 2 diabetes [27]. This indicates that treatment adherence can serve as a confounding variable when examining the effects of fructose, particularly in diabetic patients. Additionally, children are more vulnerable to excessive fructose consumption. At this developmental stage, they often lack sufficient knowledge to understand the long-term implications of high fructose intake. Consequently, the effects of dyslipidemia and decreased insulin sensitivity may accumulate, exacerbating the risks associated with excessive fructose consumption [28,29].

Fructose and uric acid

Furthermore, excessive fructose consumption is associated with elevated blood uric acid levels [15,30,31]. High uric acid levels can lead to various health issues, including kidney disease and joint inflammation. During fructose metabolism in the liver, uric acid is produced as a by-product. Excessive intake of fructose can result in the overproduction of uric acid, particularly in individuals with disorders of purine metabolism. Elevated uric acid levels have been linked to an increased risk of developing gout, a type of inflammatory arthritis.

**Figure 4:**
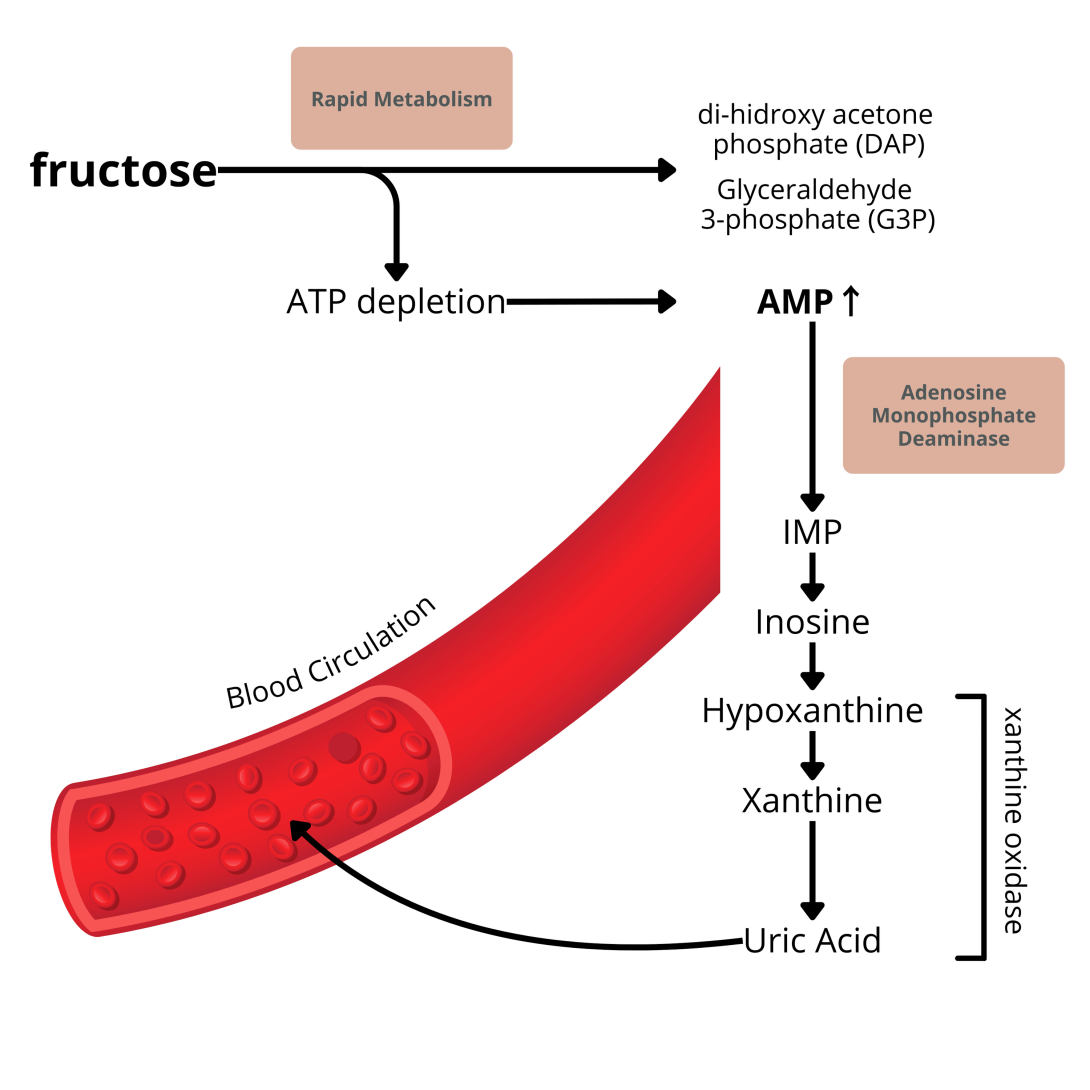
Mechanism by which fructose stimulates uric acid production AMP: adenosine monophosphate; ATP: adenosine triphosphate; IMP: inosine monophosphate

The mechanism by which fructose stimulates uric acid production is illustrated in Figure 4.

Fructose metabolism in the liver can indirectly enhance uric acid production through several biochemical reactions. When fructose is consumed in excess, it is rapidly phosphorylated to fructose-1-phosphate within liver cells, depleting ATP reserves and increasing adenosine diphosphate (ADP) levels. The elevation in ADP stimulates the enzyme adenosine monophosphate (AMP) deaminase, which catalyzes the conversion of AMP to inosine monophosphate (IMP). This IMP is then further metabolized into uric acid via the purine metabolism pathway.

Moreover, the depletion of ATP activates additional purine nucleotide degradation pathways, leading to increased production of hypoxanthine, a crucial precursor in uric acid formation [32-34]. Hypoxanthine undergoes oxidation to xanthine and subsequently to uric acid, exacerbating hyperuricemia. Additionally, fructose can indirectly elevate uric acid levels by inducing insulin resistance and oxidative stress, both of which impair uric acid excretion by the kidneys (Figure 4).

The accumulation of uric acid due to increased production and reduced excretion can lead to hyperuricemia, which is a risk factor for various diseases, including gout, chronic kidney disease, and cardiovascular disease [35].

Fructose and oxidative stress

Various studies have demonstrated that excessive fructose consumption, particularly from HFCS, can lead to oxidative stress, which triggers inflammation and contributes to disease development [20]. Oxidative stress represents an imbalance between the production of free radicals and the body’s ability to neutralize them with antioxidants. In the liver, this oxidative stress induced by excessive fructose consumption occurs as a result of an increased rate of lipogenesis [2,12].

This phenomenon has been recognized as a critical factor in the development and progression of various diseases, particularly cardiovascular and renal disorders, as it induces cellular damage and organ dysfunction. The metabolism of fructose can elevate the production of reactive oxygen species (ROS), which, if not neutralized, can cause significant damage to cells and tissues. Therefore, antioxidants play a vital role in daily health by helping to mitigate the harmful effects of oxidative stress.

**Figure 5:**
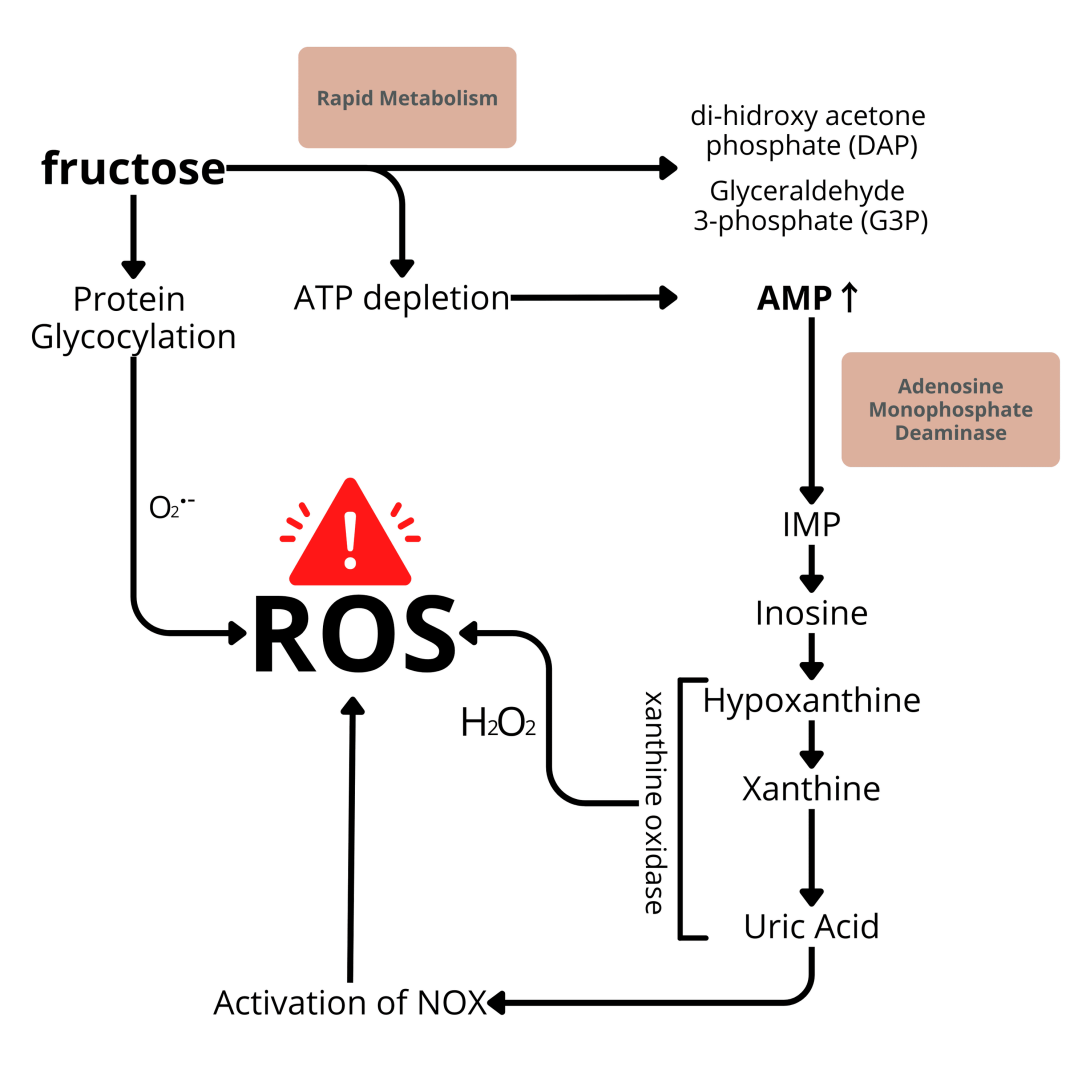
Mechanism by which fructose stimulates oxidative stress AMP: adenosine monophosphate; ATP: adenosine triphosphate; IMP: inosine monophosphate; NOX: NADPH oxidase

The mechanism by which fructose stimulates oxidative stress is illustrated in Figure 5.

As fructose is metabolized, it promotes the overproduction of ROS, such as superoxide anions, through several mechanisms. First, the rapid breakdown of fructose leads to excessive generation of nicotinamide adenine dinucleotide phosphate and acetyl-CoA, which are crucial for fatty acid synthesis. This process results in mitochondrial dysfunction and increased electron leakage from the electron transport chain, enhancing ROS production [36]. Additionally, the conversion of fructose to fructose-1-phosphate depletes cellular ATP stores, activating xanthine oxidase (Figure 5). This enzyme catalyzes the oxidation of hypoxanthine to xanthine and ultimately to uric acid, generating superoxide radicals as a by-product [37].

Elevated levels of ROS overwhelm the body’s antioxidant defense system, particularly enzymes such as superoxide dismutase and glutathione peroxidase. When ROS production surpasses the capacity of these antioxidants to neutralize them, oxidative stress ensues, resulting in cellular damage. This damage can manifest in various forms, including lipid peroxidation, protein oxidation, and DNA damage, disrupting cellular homeostasis and triggering inflammation.

Moreover, fructose-induced oxidative stress activates inflammatory pathways, particularly the nuclear factor kappa B (NF-κB) signaling pathway [37,38]. NF-κB is a transcription factor that, when activated, produces pro-inflammatory cytokines, further exacerbating tissue damage and promoting the progression of metabolic disorders such as insulin resistance, type 2 diabetes, and cardiovascular diseases.

Additionally, fructose-induced oxidative stress has been linked to endothelial dysfunction, a precursor to atherosclerosis and hypertension. The excess ROS generated from fructose metabolism can impair nitric oxide bioavailability, a key regulator of vascular tone [37,39,40]. This leads to impaired vasodilation and increased vascular stiffness, contributing to the development of cardiovascular diseases.

Overall, the metabolic consequences of fructose consumption, especially in excessive amounts, create a vicious cycle where oxidative stress promotes inflammation, cellular damage, and organ dysfunction, particularly in the liver, kidneys, and cardiovascular systems. This makes reducing excessive fructose intake and enhancing antioxidant defenses crucial strategies for mitigating the harmful effects of oxidative stress.

## Conclusions

The discussion emphasizes that high fructose consumption adversely affects human health, particularly concerning obesity, hyperglycemia, type 2 diabetes, and elevated uric acid levels. It is essential to limit daily fructose intake, particularly from added sugars in processed foods and beverages, and implementing government regulations could be beneficial. Understanding the liver’s critical role in the negative effects of excessive fructose metabolism is vital. Additionally, antioxidant supplementation and lifestyle modifications aimed at reducing fructose intake can help alleviate the oxidative stress and inflammation associated with high fructose consumption.

## References

[1] Mai BH, Yan LJ (2019). The negative and detrimental effects of high fructose on the liver, with special reference to metabolic disorders. Diabetes Metab Syndr Obes.

[2] Camici M, Allegrini S, Tozzi MG (2018). Interplay between adenylate metabolizing enzymes and AMP-activated protein kinase. FEBS J.

[3] Cho IJ, Oh DH, Yoo J (2021). Allopurinol ameliorates high fructose diet induced hepatic steatosis in diabetic rats through modulation of lipid metabolism, inflammation, and ER stress pathway. Sci Rep.

[4] Dekker MJ, Su Q, Baker C, Rutledge AC, Adeli K (2010). Fructose: a highly lipogenic nutrient implicated in insulin resistance, hepatic steatosis, and the metabolic syndrome. Am J Physiol Endocrinol Metab.

[5] DiStefano JK, Shaibi GQ (2021). The relationship between excessive dietary fructose consumption and paediatric fatty liver disease. Pediatr Obes.

[6] Dornas WC, Cardoso LM, Silva M (2017). Oxidative stress causes hypertension and activation of nuclear factor-κB after high-fructose and salt treatments. Sci Rep.

[7] Dornas WC, de Lima WG, Pedrosa ML, Silva ME (2015). Health implications of high-fructose intake and current research. Adv Nutr.

[8] Douard V, Ferraris RP (2013). The role of fructose transporters in diseases linked to excessive fructose intake. J Physiol.

[9] Dyer J, Wood IS, Palejwala A, Ellis A, Shirazi-Beechey SP (2002). Expression of monosaccharide transporters in intestine of diabetic humans. Am J Physiol Gastrointest Liver Physiol.

[10] Federico A, Rosato V, Masarone M, Torre P, Dallio M, Romeo M, Persico M (2021). The role of fructose in non-alcoholic steatohepatitis: old relationship and new insights. Nutrients.

[11] Ferraris RP, Choe JY, Patel CR (2018). Intestinal absorption of fructose. Annu Rev Nutr.

[12] Francisqueti FV, Santos KC, Ferron AJ (2016). Fructose: toxic effect on cardiorenal risk factors and redox state. SAGE Open Med.

[13] Gaspers LD, Thomas AP (2010). Mechanisms underlying fructose-induced oxidative stress in the cytosol and mitochondria. Biophys J.

[14] Hu G, Xu L, Ito O (2023). Impacts of high fructose diet and chronic exercise on nitric oxide synthase and oxidative stress in rat kidney. Nutrients.

[15] Huang Z, Xie N, Illes P (2021). From purines to purinergic signalling: molecular functions and human diseases. Signal Transduct Target Ther.

[16] Johnson RJ, Perez-Pozo SE, Sautin YY (2009). Hypothesis: could excessive fructose intake and uric acid cause type 2 diabetes?. Endocr Rev.

[17] Johnson TA, Jinnah HA, Kamatani N (2019). Shortage of cellular ATP as a cause of diseases and strategies to enhance ATP. Front Pharmacol.

[18] Koniah E, Sarnianto P (2021). Faktor penentu keterkendalian glukosa darah pada pasien diabetes melitus tipe II di rumah sakit Bina Husada Cibinong. Syntax Literate.

[19] Lee DH, Lee JU, Kang DG, Paek YW, Chung DJ, Chung MY (2001). Increased vascular endothelin-1 gene expression with unaltered nitric oxide synthase levels in fructose-induced hypertensive rats. Metabolism.

[20] Lee H, Kim E, Shin EA (2022). Crosstalk between TM4SF5 and GLUT8 regulates fructose metabolism in hepatic steatosis. Mol Metab.

[21] Li X, Joh HK, Hur J (2023). Fructose consumption from different food sources and cardiometabolic biomarkers: cross-sectional associations in US men and women. Am J Clin Nutr.

[22] Liang RJ, Taylor S, Nahiyaan N (2021). GLUT5 (SLC2A5) enables fructose-mediated proliferation independent of ketohexokinase. Cancer Metab.

[23] Lustig RH (2020). Ultraprocessed food: addictive, toxic, and ready for regulation. Nutrients.

[24] Mayes PA (1993). Intermediary metabolism of fructose. Am J Clin Nutr.

[25] Muriel P, López-Sánchez P, Ramos-Tovar E (2021). Fructose and the liver. Int J Mol Sci.

[26] Nakagawa T, Johnson RJ, Andres-Hernando A, Roncal-Jimenez C, Sanchez-Lozada LG, Tolan DR, Lanaspa MA (2020). Fructose production and metabolism in the kidney. J Am Soc Nephrol.

[27] Ngo AN (2020). Hidden sugar and its bitter obstacles for the wellbeing of consumers. Mark Global Dev Rev.

[28] Nomura K, Yamanouchi T (2012). The role of fructose-enriched diets in mechanisms of nonalcoholic fatty liver disease. J Nutr Biochem.

[29] Nomura N, Verdon G, Kang HJ (2015). Structure and mechanism of the mammalian fructose transporter GLUT5. Nature.

[30] Johnson RJ, Nakagawa T, Sanchez-Lozada LG (2013). Sugar, uric acid, and the etiology of diabetes and obesity. Diabetes.

[31] Shi YN, Liu YJ, Xie Z, Zhang WJ (2021). Fructose and metabolic diseases: too much to be good. Chin Med J (Engl).

[32] Stanhope KL, Havel PJ (2009). Fructose consumption: considerations for future research on its effects on adipose distribution, lipid metabolism, and insulin sensitivity in humans. J Nutr.

[33] Stricker S, Rudloff S, Geier A, Steveling A, Roeb E, Zimmer KP (2021). Fructose consumption-free sugars and their health effects. Dtsch Arztebl Int.

[34] Suwannakul N, Armartmuntree N, Thanan R (2022). Targeting fructose metabolism by glucose transporter 5 regulation in human cholangiocarcinoma. Genes Dis.

[35] Tappy L, Lê KA (2010). Metabolic effects of fructose and the worldwide increase in obesity. Physiol Rev.

[36] Todoric J, Di Caro G, Reibe S (2020). Fructose stimulated de novo lipogenesis is promoted by inflammation. Nat Metab.

[37] Velázquez AL, Vidal L (2021). Significant sugar-reduction in dairy products targeted at children is possible without affecting hedonic perception. Int Dairy J.

[38] Weng Y, Zhu J, Chen Z, Fu J, Zhang F (2018). Fructose fuels lung adenocarcinoma through GLUT5. Cell Death Dis.

[39] White JS, Hobbs LJ, Fernandez S (2015). Fructose content and composition of commercial HFCS-sweetened carbonated beverages. Int J Obes (Lond).

[40] Zhang DM, Jiao RQ, Kong LD (2017). High dietary fructose: direct or indirect dangerous factors disturbing tissue and organ functions. Nutrients.

